# Scoring system for assessing the symmetry of tooth development in mixed dentition

**DOI:** 10.4317/jced.60789

**Published:** 2023-09-01

**Authors:** Reinhard E. Friedrich, Moritz Schön

**Affiliations:** 1 Professor Doctor, Department of Oral and Craniomaxillofacial Surgery, Eppendorf University Hospital, University of Hamburg, Hamburg, Germany; 2 Postgraduate dentist, Department of Oral and Craniomaxillofacial Surgery, Eppendorf University Hospital, University of Hamburg, Hamburg, Germany

## Abstract

**Background:**

Human teeth develop sequentially and symmetrically in both halves of the jaws. Disorders of tooth development and eruption can be local or generalized. The symmetry comparison of tooth development is an essential measuring point to assess dentition. The aim of this study was the development of an easy-to-apply score that represents the tooth development of one side of the jaw to carry out side-specific development comparisons.

**Material and Methods:**

The stages of development and the state of health of the teeth were determined on orthopantomograms of 59 healthy children and adolescents applying acknowledged developmental standards of teeth. The individual stages of tooth development on one side of the jaw were combined into a numerical score. The sum score of each jaw side was compared.

**Results:**

The dental developmental score reveals the side differences of tooth development are small in children and adolescents with mixed dentition (n.s.). The change of teeth starts earlier in females.

**Conclusions:**

The presented score enables an easily applicable examination of the symmetry of tooth development in mixed dentition. Potential applications of the Score are to examine the influence of unilaterally manifest dento-skeletal developmental disorders on the change of teeth and the influence of deviating individual tooth development on neighboring teeth on one side.

** Key words:**Dentition, development, symmetry, permanent teeth, deciduous teeth, oral health.Dentition, development, symmetry, permanent teeth, deciduous teeth, oral health.

## Introduction

Dental health examinations are essential components of maintaining health care and assessment of the physical condition. Already in childhood, far-reaching assumptions or even diagnoses about an individual can often be drawn from assessing dental development and findings. The assessment of tooth formation and eruption is of fundamental importance in dental science and practice (1,2). Considering the multitude of processes involved in tooth formation and eruption, the time corridor is narrow for the expected eruption of symmetrically positioned and shaped teeth (3,4). Therefore, in each jaw a largely symmetrical development of antimeric teeth is assumed (5). In the biological framework, tooth development is characterized by a largely symmetrical change from deciduous to permanent dentition. Tooth change takes place over a considerably long phase of life and shows individual variability of the total duration (1,2). External influences (caries, trauma) can affect the individual tooth status. Deviations from the symmetry of the dentition are often the first indication of complex dental disorders (4). In order to also capture discrete influences on the symmetry of the dentition (6), the assessment of the natural variability of the phenomenon is of some value. The symmetry of tooth replacement can be examined for individual teeth and tooth groups in children and adolescents, e.g., by oral investigation or on radiographs depicting the whole dentition (1,2). The present study examines the suitability of the body-side-specific summation of radiologically assessed developmental stages of teeth into a score as an aid to checking symmetry of dental development. The purpose of the study is to develop a simply applicable test standard for comparing the current status of tooth development from side to side. Application of this score may facilitate identification of unilaterally manifest tooth development and eruption disorders.

## Material and Methods

Patients and radiographs. Fifty-nine orthopantomogram (OPG) images of the jaws taken from 59 patients were selected (study group, SG) for investigating dental health and development.

The anonymized radiographs were obtained from patient files of the Radiology Department, Dental Clinic (Head: C. Scheifele, DMD). Only OPGs of patients were evaluated, which were prepared for clarification of a potential orthodontic treatment. Patients with known syndrome, history of maxillofacial surgery, or known facial trauma were excluded from the selection process. Exclusion of OPGs from evaluation for technical reasons was due to projection-related divergences such as shadowing, overlapping, distortion or a tooth axis deviation perpendicular to the projection plane. The evaluation considered each individual tooth (position).

The analysis was carried out for the right and left sides of the jaw considering 16 evaluation criteria in 59 people. In 35 male and 24 female patients, each tooth category was available in 118 assessments on the right and left side respectively (males: 70, females: 48). The age of the patients ranged from 3 to 18 years (ys). The mean age of the group was 10.32 ys (females: 12 ys; males: 9.17 ys). Fédération Dentaire Internationale (FDI) tooth code according to Viohl’s proposal (7) was used for tooth numbering and graphical representation of a patient’s finding, that is, the teeth were numbered quadrant-wise starting with the value “1” for the central incisor, from anterior to posterior. The quadrants were numbered from 1 to 4, starting with the upper right quadrant and following the clockwise direction. Accordingly, the quadrants for the teeth of the first dentitions were identified with the values 5-8. The indication of the quadrant and the individual value of the tooth result in a numerical value consisting of two digits for each tooth and allow the unambiguous identification of each tooth of the respective dentition. This dental identification was used for the calculations.

**Figure 1:**
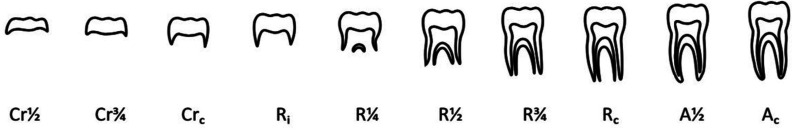
Classification of the developmental stages of teeth according to Gleiser and Hunt (16), modified according to Köhler et al. (17) ( Cr = crown, R = root, A = apex, lowered “c” = complete).

**Figure 2:**
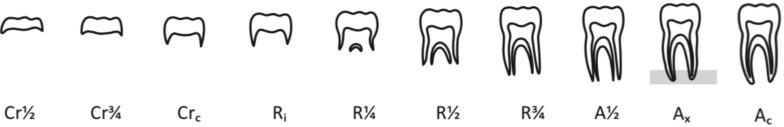
Modified dental growth stage classification of this study.

Dental examination parameters. A total of 3,068 teeth/tooth positions were expected, considering potential 32 permanent teeth and 20 deciduous teeth of 59 individuals. Ten of 16 classification criteria addressed dental growth stage (Fig. 1,2). Emphasis was placed on examining the calcification of the teeth (10 developmental stages, i.e., crown and root growth, and morphology of the apex). The numerical individual values of the development of the teeth on one side of the jaw or one side of the body collected for an individual were combined into a total or sum score. This individual score was used to examine the differences in the dental development side-by-side of the single or both jaws.

In addition, the number of teeth/missing teeth, decayed and filled teeth, presence of tooth germs, and impacted teeth were considered. A total of 16 parameters were evaluated for each tooth individually. Permanent and deciduous teeth were considered separately for evaluation.

If a tooth was classified ‘missing, germinated or partially resorbed’, the tooth was not considered in the sum score of dental development. A focus of the study was is the examination of the symmetry of the stages of tooth development comparing the added developmental stages of the jaw halves. X-rays were read individually by each examiner and questionable findings were decided by consensus.

The parameters were defined as follows:

1. Missing tooth. If neither a clue for a tooth-like structure nor a tooth follicle was recognizable on OPG, a tooth was considered missing (8).

2. Retained tooth. A tooth was classified as retained if the tooth was located within the bone, below the line of occlusion, and the maximum physiological eruption time was exceeded by more than 1.5 years. A tooth was also classified as retained if the tooth was not in an orthograde eruption position, preventing passage into the oral cavity (9).

3. Caries. A tooth was considered carious (decayed) if a radio-translucent area was identified that was not filling-related. The radio-translucent area was surveyed around the tooth crown with transition to the tooth root. The radiological diagnosis of caries is limited due to the recording technique of the OPG. Therefore, only areas with unambiguous dental radio-transparencies at typical sites and of characteristic shape were considered (8).

4. Fillings. A tooth was evaluated as restored if the radiograph showed one or more clearly demarcated opaque lines, which were interpreted as preparation margins, and within this boundary there was a radio-opacity deviating from the radio-transparency of the crown or root. This radio-opaque change within the tooth outline could be judged to be metal-tight, equivalent to or deviating from enamel or dentin density and was summarily recorded as “filling” when detected regardless of the number of findings per tooth (8).

5. Tooth development stages. Ten growth stages were classified according to the stages of tooth crown development, root mineralization and conFiguration of the apex proposed by Gleiser and Hunt (10), modified by Köhler *et al*. (11). The more a tooth is calcified in apical direction, the greater the numerical value (Figs. 1,2).

Distinct modification of the referred growth staging systems was made to ensure easily reproducible results: The growth stage Rc includes roots with “approximately full root length with diverging root canal”. This item often cannot be reliably located on OPG due to the limited resolution of structures and variable projections of root regions in this survey image and was therefore omitted. Instead, the category A1/2 “full root length, parallel root canal” was supplemented by the further differentiation “apex open”. For the growth stage Ac “full root length, converging root canal”, the classification criterion was supplemented by the addition of “apex closed”. The stage Ax “full root length, apex not assessable” does not occur in the modified classification of Gleiser and Hunt (11) and was added here to enable a distinction to be made between the resolution- and image-related limits of X-ray findings and superimpositions and projections of teeth and other structures in the field of view. With this criterion, it is also possible to evaluate teeth that can be assessed with such limitations of radiological presentation and have reliably completed root development.

6. Germination. A tooth developmental stage that did not yet correspond to the criteria applicable for first stage according to Gleiser and Hunt (10) (crown formation) but could already be classified as osseous roundish/oval shading of odontogenic origin, was classified as germinal, i.e. no evidence of tooth crown-like radio-opacity in a radio-transparent region of a jaw in a typical position of a developing tooth.

7. Partially resorbed deciduous tooth. The evaluation includes the classification of the developmental stages of deciduous and permanent teeth. Since the roots of deciduous teeth are subject to resorption by odontoclasts during the natural tooth change process, assignment to a growth stage may not be possible. In this case, presence of partially resorbed deciduous tooth was recorded and evidence/exclusion of caries.

Scoring of tooth development. The individual parameters were assessed and registered in binary form: 0 = does not apply; 1 = does apply. However, dental development stage of each tooth (Cr ½ to Ac) was further specified in a numerical code according to the development stage classification (Fig. 1).

Only one of the development stages in the individual case could apply. This means that one of the 10 values applied in the individual case and was scaled in an ascending numerical sequence from 1-10 as the tooth development progresses. The higher the value of the score, the more advanced the tooth development was. If, for example, the fourth development stage applied (Ri = 1) (Fig. 2), the score of the tooth was evaluated as 4.

The classification allowed the comparison of developmental stages of individual teeth and tooth groups. The t-test was used to calculate the growth stages. For further calculations, following the individual assessment, the summarized developmental stages of a quadrant were registered for symmetry evaluation of a jaw and/or body side.

Statistics. Data were recorded in an Excel™ sheet (Microsoft Corp., Redmond, WA, USA. Using SPSS™, version 25 (IBM Corp., Armonk, New York, USA). Cross tabulations were created based on the data collection and significance was determined using the Chi-Square test. A difference *p*<0.05 was recorded as significant. Initially, the right and left jaw sides within the SG were compared, i.e., the sum values of the 1st and 4th quadrants with those of the 2nd and 3rd quadrants. A crosstab was created for the comparing dental findings of each tooth. Values were analyzed for frequency and symmetry within an individual. Each value was considered separately for each side of the jaw and a side-by-side comparison was made.

Ethics. All procedures of this study were performed following the ethical standards of the institutional and/or national research committee and with the 1964 Declaration of Helsinki and its later amendments or comparable ethical standards. Before analysis, data were anonymized, and the investigators studying the radiographs were blinded to diagnosis and individual identity. The investigations into anonymized data were performed following Hamburgisches Gesundheitsdienstgesetz (Hamburg Health Services Act). This law regulates the permission to use anonymized patient data for scientific purposes (§11). This type of investigation does not require the approval of the local ethics committee. The examinations are part of a scientific thesis to fulfill the requirements for the attainment of a doctorate (in dentistry) at the faculty of medicine of the university (MS).

 

## Results

A total of 889 teeth were missing in the study group (28.9%). A total of 2,179 teeth were evaluated.

Deciduous teeth. In the study group, 752 of the potentially 1180 deciduous teeth were missing (missing teeth by body side (right/left): males 183/179; females 195/195 (*p*>0.05, n.s.; evaluable: 428 (males 338, females 90)). However, comparison of number of missing deciduous teeth considering sex disclosed the higher number of missing teeth in females (*p*<0.001).

Decayed teeth of males showed no lateral preference (right: 23, left 26, *p*>0.05, n.s.) Decayed teeth were less common in females (right: 1, left 0, n.s.). However, the differences in the number of decayed teeth in the gender comparison were highly significant: Males had significantly more decayed teeth (*p*<0.001).

Of evaluable deciduous 338 teeth in males, 15 showed fillings on the right side and 14 on the left side. In females (n=90), 3 teeth were filled on the right and 5 on the left. The number of dental fillings showed no preference of gender (*p*=0.926).

Applying of the classification criteria of the dental growth stages, only 36/214 (right) and 40/214 deciduous teeth could be assessed (males: 35 (right) and 37 (left); females: 1 (right) and 3 (left), (*p*>0.05, n.s). The considerably higher number of teeth evaluated in terms of tooth development stages in males is due to the higher number of missing teeth in females (*p*<0.001).

Due to the age-related high proportion of patients with a physiological change of teeth, assessment ability of developmental stages was restricted to only a minority of deciduous teeth. The total number of teeth showing root resorption was the same on both sides of the body considering sex (*p*>0.05). The study shows symmetrically distributed values of teeth evaluable for this parameter by side for males (right: 132, left: 134) and females (right: 44, left: 42). However, more teeth were evaluable in males (*p*<0.001). This finding is complementary to the more advanced tooth change in females (vide supra).

Permanent teeth. A total of potentially 1888 permanent teeth (59x32) was expected (944 teeth on each jaw side). However, 137 permanent teeth were missing: right side 67, left side: 70 (males: 87 of 1120 (7.7%), females: 50 of 768 (6.51%) allowing dental development and health assessment in 1751 teeth (males: 1033; females: 718).

On the right side of males, 41 of 560 teeth were missing, in females 26 of 384 (left side, males: 46/560, left side, females: 24/384; *p*>0.05, n.s.).

Decayed teeth were diagnosed in 19 teeth of each side (males: 2x8; females: 2x11, *p*>0.05, n.s.).

The difference in filled teeth was not significant between the right and left sides of the jaw (20/852 and 17/851, calculations excluded tooth germs). Taking gender into account, 7/499 (males) and 13/353 (females) teeth were registered as filled on the right side and 7/ 496 (males) and 10/355 (females) on the left side, respectively (*p*>0.05, n.s.).

The development stages of the teeth, assessed according to 10 ranks, were applied to 499 (right) and 496 (left) teeth of males (96.1% and 96.5%, resp.). For females, scoring was applied at 353 (right) and 355 (left) (both: 98.6%). The criterion “tooth germ” was met in males in 38 cases (right: 20, left 18, *p*>0.05, n.s.). In females, 5 tooth germs were registered on both sides. Overall, the differences in scoring of dental developmental stages were not significant considering body side and the individual tooth comparison (Tables 1,2).

There were no significant differences in the overall side comparison as well as in the individual tooth comparison (T-test, *p*>0.05). The number of teeth that were similar in growth stage on both sides of the jaw could nevertheless differ significantly with regard to the distribution of growth stages. Results are summarized in  Table 1- Table 3.

**Table 1:**
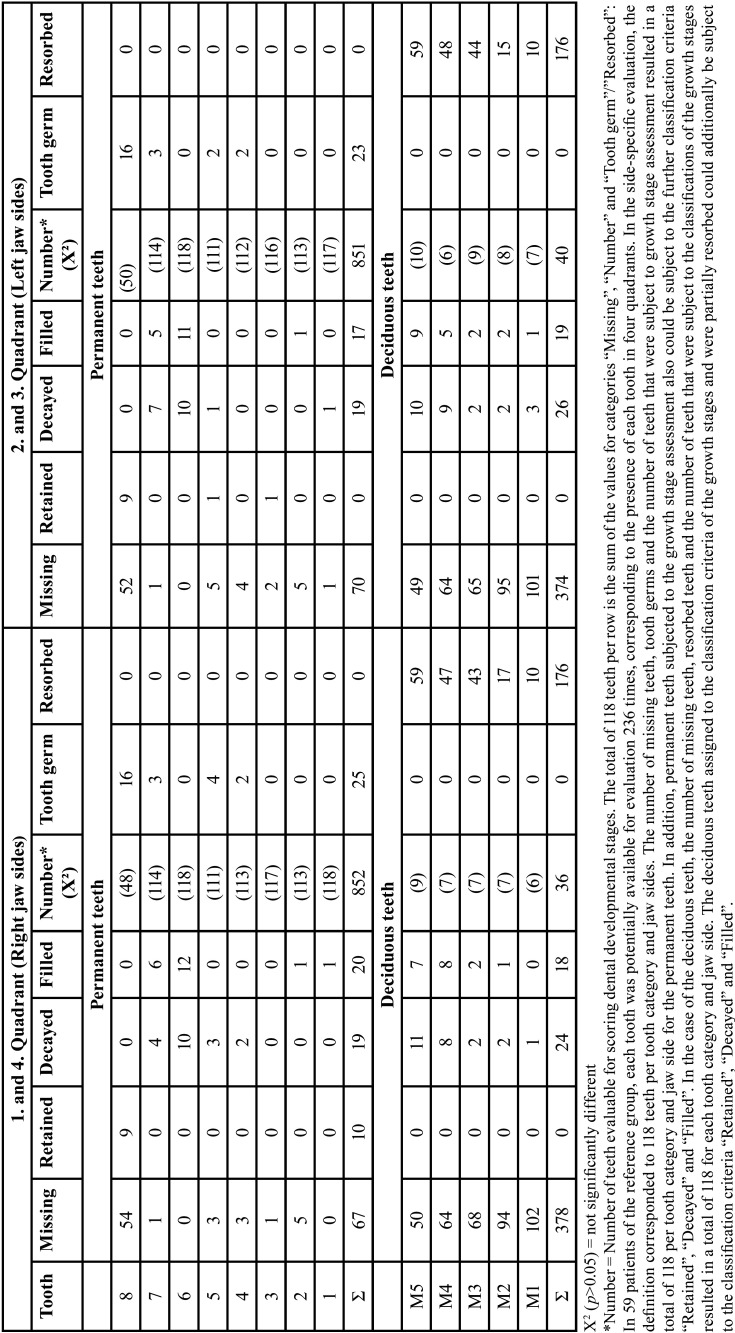
Number of deciduous and permanent teeth of the study group. Left and right sides of the jaws are compared (right side = 1. and 4. quadrant, left = 2. and 3. quadrant). Teeth numbered according to FDI (7).

**Table 2:**
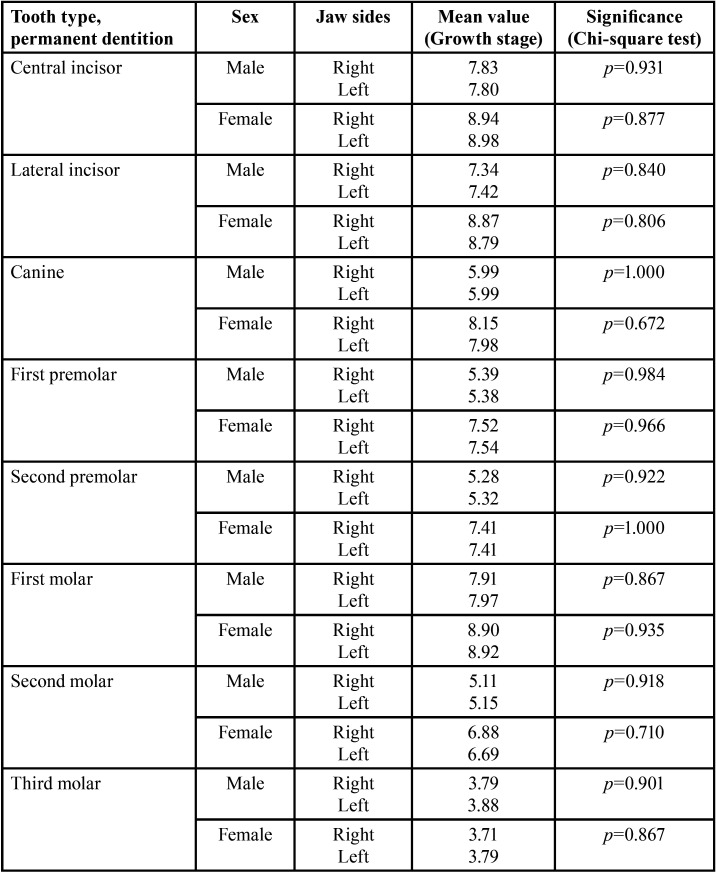
Comparison of study group’s growth stages of permanent teeth considering sex (T-tests for independent samples).

**Table 3:**
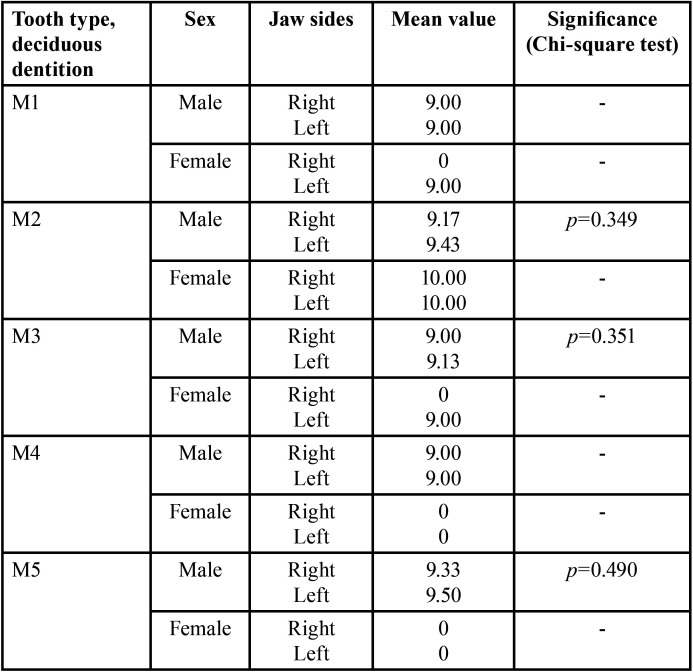
Comparison of study group’s growth stages of deciduous teeth considering sex (T-tests for independent samples).

## Discussion

The examination shows the symmetry of tooth root development and tooth eruption in children and adolescents. The summary of dental developmental stages of one half of the jaw enables side-by-side comparison of the dentition status of functional units within one jaw and between body sides. The method is suiTable for providing a calculation basis for the quantified assessment of developmental differences in the case of local developmental disorders of the teeth that are limited to one side.

When examining time-related tooth eruption anomalies, the focus usually is on the delayed emergence of the tooth/teeth (4). For tooth eruption, there is a relatively wide time corridor in which emergence can be expected (1,2). Radiological examinations are an essential tool for diagnosing disorders of tooth development and eruption (12). In clinical and scientific examination, assessment of dentition development applies to the single tooth development in comparison to the antimeric, in other words, the symmetry of tooth eruption is assumed - within the biological variability of a few months. The assessment criteria also apply to tooth eruption in diseases, which are known to be associated with an influence on tooth eruption (12). Causes of impaired tooth development can be local differentiation disorders, but also syndromes that are associated with a general developmental disorder concerning the dentition. In the case of local (and sporadic) causes the asymmetry of tooth development is often the indication for advanced diagnostics. Syndromic disorders affecting teeth do not necessarily present bilaterally symmetrical aberrations of tooth development (13). In addition, a syndromic disease can result in tumor-associated jaw deformities, which in combination of tooth malposition and adjacent soft tissue tumor prevent tooth eruption (14). On the other hand, a tumor-like vascular malformation of the jaw developed on one side of the face can accelerate tooth eruption (15). For assessing dentition in diseases potentially affecting dental development, the score proposed here offers an easy-to-use method to evaluate the overall dental developmental stage and oral health in an individual side-by-side comparison. Furthermore, the method can be used to assess the influence of a disease manifesting on one side of the jaw(s) on dental development. A widely available imaging technique in dentistry with low radiation exposure of the individual can be used for these purposes.

Limitations of study. One source of imaging error (OPG) is due to incorrect positioning of the patient in the device must be considered and cannot be excluded. The influence of imaging errors due to incorrect positioning, in addition to the known technical-physical deficiencies of the OPG’s limitations in the precision of imaging teeth, highlights the restricted interpretation accuracy of spatial dental relationship due the trough-shaped focus of the x-rays to generate the two-dimensional panoramic image of an arched bone (16-18). This limitation also applies to digitally reformatted OPGs from computerized tomographic images of the jaws (19).

Aplasia of teeth could not be confidently differentiated in this retrospective study and was therefore scored as ’missing tooth’ only. A tooth was considered missing if no tooth anlage was seen in the expected tooth position in correlation to the normal developmental stage.

Interpreting the low total number of decayed and restored teeth in the study group, the low average age of the SG and consequent relatively short exposure of the teeth to the oral cavity has to be considered.

For comparability of dental health results, the DMFT index was used in the above quoted comparative studies. The results of the present study are not presented using the DMFT index because, first, the DMFT index includes a clinical assessment of the dentition, secondly, the third molars are not included in the index and, finally, the side-specific evaluation of dental findings is not reflected in the index.

The levels of root resorption of deciduous teeth were used for forensic age estimation (20). In that study, the resorption stages of the teeth were assessed, except for the incisors, which were difficult to examine in detail due to the projection geometry of the OPG (17). In the study presented here, only the detection or absence of root resorption of deciduous teeth was recorded, taking into account the projection technique and the target volume recorded (focal trough). The results presented here show that the tooth change is symmetrical regarding the resorption of the predecessors, which enabled the permanent teeth to erupt.

## Conclusions

The development and eruption of teeth occurs during a considerably long period of time. Disorders of tooth development and/or tooth eruption can relate to the entire dentition, groups of teeth or individual teeth. In order to obtain more precise data for the assessment of the phenomenon in the case of local tooth development disorders, in particular for the assessment of the neighboring teeth or even one side of the jaw, an easy-to-use index for the symmetry comparison of the current tooth development is helpful. This study shows that an easy-to-use scoring system derived from well-known tooth development staging system enables the symmetry comparison of tooth development. With this, for example, in the case of a disorder with local impact on dental development and emergence, radiologically detectable differences of tooth development and eruption can be summarized, coded and compared side by side.
